# System of combined psychotherapeutic and physical rehabilitation of children with cyber addiction

**DOI:** 10.1192/j.eurpsy.2023.352

**Published:** 2023-07-19

**Authors:** B. Mykhaylov, O. Kudinova, I. Shestak

**Affiliations:** ^1^National Health Care University named Shupik, Kyiv; ^2^Kharkiv Medical Academy Postgraduate Education, Kharkiv; ^3^Lviv State University of Physical Culture after Ivan Bobersky, Lviv, Ukraine

## Abstract

**Introduction:**

Contemporary children practically live in cyberspace, constantly using various devices,and many of them have an addiction that leads to violations of their mental and physical formation.

**Objectives:**

Based on the mental and the physical examination of children suffering on cyber addiction with the sings of clinical manifestations determine the targets and form of ccmbined correctional rehabilitation system.

**Methods:**

The clinical investigation based on psychiatric examination with the narrative motivation interview,psychological examination by Eysenck “extra/introversion”test and physical examination with special attention to the musculoskeletal system.

**Results:**

The randomized investigation of childhood population was performed.198 children of different age (7 – 14 years old) were examined.72 (36.4%) of them showed significant signs of cyber addiction with a narrowing of interests, a reduction in social ties, and a decrease in school performance. They spent from 3 to 10 hours on gadgets per day.140 (71%) of them, also showed signs of fubbing, that is, the priority of telephone communication over direct communication with the interlocutor.The significant results of Eysenck test examination were following: 126(64%) of them were introverts and 72(36%) of them were extraverts. During the physical examination,91 (46%) of these children had different spine and musculoskeletal disturbances.In the group of addictional children the vast majority of subjects, 87%, have characteristic defects of the musculoskeletal system. The most symptomatic in the diagnosis of cyberaddiction and phubbing of school-age children are: round back, stooping, scoliotic posture and types of lateroflexion.The severity of cyber addiction and the degree of musculoskeletal disorders were inversely proportional. The results served as the basis for the development of a combined system of psycho-physical rehabilitation. The psychotherapy based on CBT according to A. Beck, modified for children’s in concordance with specialized program “Vertebra-Dinamik” to correct defects of the musculoskeletal system. The basis of the corrective “Vertebra-Dinamik” technique includes a comprehensive approach to the use of isometric and statodynamic exercises of moderate intensity with elements of relaxation. Approbation of the developed system showed its high( 80%) and moderate(20%) efficiency.

**Conclusions:**

To successfully overcome cyber addiction and its negative consequences, the most optimal system is the complex application of psychotherapeutic and physical rehabilitation methods.

**Disclosure of Interest:**

None Declared

